# Drying of tundra landscapes will limit subsidence-induced acceleration of permafrost thaw

**DOI:** 10.1073/pnas.2212171120

**Published:** 2023-02-13

**Authors:** Scott L. Painter, Ethan T. Coon, Ahmad Jan Khattak, Julie D. Jastrow

**Affiliations:** ^a^Climate Change Science Institute and Environmental Sciences Division, Oak Ridge National Laboratory, Oak Ridge, TN 37831; ^b^Environmental Science Division, Argonne National Laboratory, Lemont IL 60439

**Keywords:** permafrost, active layer, climate change, thermokarst

## Abstract

We used a model for permafrost hydrology informed by detailed measurements of soil ice content to better understand the potential risk of abrupt permafrost thaw triggered by melting ground ice, a key open question associated with permafrost response to a warming Arctic. Our spatially resolved simulations of a well-characterized site in polygonal tundra near Utqiaġvik, Alaska, agree well with multiple types of observations in the current climate. Projections indicate 63 cm of bulk subsidence from 2006 to 2100 in the strong-warming Representative Concentration Pathway 8.5 climate. Permafrost thaw as measured by the increase in active layer thickness (ALT)—the thickness of the soil layer that thaws each summer—is accelerated by subsidence, but the effect is relatively small. The ALT increases from the current-day value of approximately 50 cm to approximately 180 cm by 2100 when subsidence is included compared to about 160 cm when it is neglected. In these simulations, previously identified positive feedbacks between subsidence and thaw are self-limiting on decadal time frames because landscape runoff and increasing evapotranspiration result in drier tundra with weaker surface/atmosphere coupling. These results for a tundra site that is representative of large swathes of the Alaska North Slope suggest that subsidence is unlikely to lead to abrupt thaw over large areas. However, subsidence does have significant effects on the hydrology of polygonal tundra. Specifically, subsidence increases landscape runoff, which helps maintain streamflow in the face of increased evapotranspiration but also causes drier tundra conditions that could have deleterious effects on sensitive Arctic wetland ecosystems.

## Significance

Topography change caused by melting ice in Arctic soils has the potential to accelerate permafrost thaw and trigger abrupt and large-scale change in the Arctic. We extended a site-scale permafrost thermal hydrology model to represent ground subsidence and combined it with new and existing data from a well-characterized tundra site to better understand the consequences of thaw subsidence in a warming Arctic. Our spatially resolved simulations of a representative carbon-rich tundra site indicate that subsidence will not accelerate permafrost thaw significantly and cause abrupt permafrost thaw over large areas. However, thaw subsidence will likely lead to more runoff and significantly accelerate drying of the tundra landscape in a warming climate with important effects on sensitive Arctic ecosystems.

Arctic permafrost regions are warming significantly faster than the rest of the Earth and are already experiencing widespread environmental change (1). These trends are expected to continue for decades even in optimistic scenarios for future anthropogenic carbon releases to the atmosphere. As a result, much of the approximately 1,000 Pg of organic carbon currently stored in the upper 3 m of soils in Arctic permafrost landscapes (2–4)—significantly more than is currently in the atmosphere—is vulnerable to thawing and potential release to the atmosphere as greenhouse gases. The fate of that carbon as well as sensitive ecosystems and economically important infrastructure in the Arctic depends critically on the thermal hydrologic response of permafrost to Arctic warming.

Many of the key uncertainties about the response of Arctic permafrost to a warming climate are related to the effects of excess ground ice (1, 2, 5). In reference to permafrost soils, “excess ice” refers to ice in amounts that exceed the porosity of the unfrozen soil. Upon melting of excess ice, soils lose the structural support of that ice and consolidate until the structurally competent porosity is reached, causing ground subsidence. Because excess ice is typically unevenly distributed in ice lenses and wedges, thaw-induced subsidence is often nonuniform and can alter topography, a process termed thermokarst formation (6). Thermokarst formation can have important effects on landscape hydrology (7, 8) and has been observed to create localized ponding and focused flow, potentially triggering positive feedbacks and accelerating permafrost thaw (2, 5). Inventory-based analyses (5) highlight the potential global relevance of abrupt thaw. Based on those and similar studies, the Intergovernmental Panel on Climate Change assessed thermokarst-induced abrupt thaw as having potential to drive rapid and large-scale environmental change but identified significant uncertainties and assigned only *medium confidence* to that assessment because models required for quantitative projections are limited (1). Thermokarst-accelerated thaw is not represented in the current generation of Earth system models, although the thermal effects of excess ice are occasionally included without thermokarst formation (9).

**Fig. 1.**
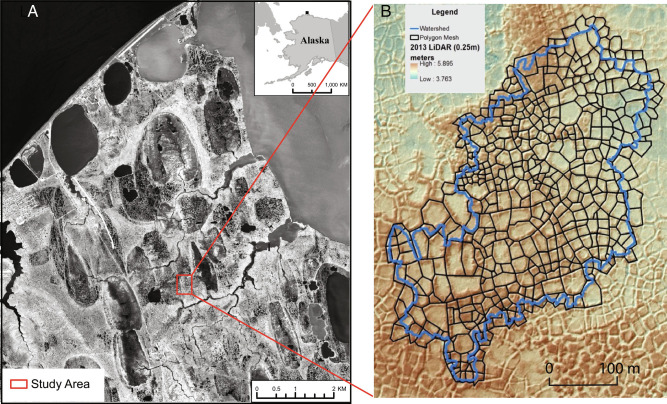
Location of the study area near Utqiaġvik, AK. All simulation results shown here are for the catchment outlined in blue in panel *B*. Panel *A* was adapted from Atchley et al. ref. 14.

Here, we aim to significantly reduce major uncertainties about the effects of thermokarst formation on permafrost degradation using a new version of a cryohydrology model informed by new measurements of excess ice content and existing data from the decade-long Next-Generation Ecosystem Experiment—Arctic program. We focus on polygonal tundra as an important type of landscape that is widespread in lowland continuous permafrost regions, rich in soil organic carbon, and vulnerable to thermokarst formation and abrupt thaw (10). In polygonal tundra, vertically oriented ice wedges express on the surface in polygonal patterns (see, for example, the digital elevation model in Fig. 1*B*). Nonuniform subsidence caused by ice-wedge melting has been widely observed to cause ice-wedge polygons to transform from a characteristic microtopography with shallow depressions in the polygon centers to a microtopography with elevated centers and well-defined troughs on the edges (7, 11–13). Those two major polygon types have very different hydrological response. In particular, the troughs that form the sides of high-centered polygons are effective in draining the landscape, while shallow depressions in the centers of low-centered polygons retain water and impede landscape drainage (7). Those differences in landscape drainage imply different inundation patterns and soil moisture states with important consequences for surface ecosystems, soil biogeochemical processes, and atmosphere–surface interactions.

Preliminary steps have been taken to include the process of thermokarst formation in models of polygonal tundra, but the process representations remain highly idealized. Nitzbon et al. (10) modified the CryoGrid 3 model (15) to include thermokarst formation and showed that the process has potential to greatly accelerate thaw but is highly sensitive to landscape drainage efficiency. CryoGrid 3 is not spatially resolved and instead uses three representative vertical columns to model landscape dynamics. Assessment of landscape runoff and drainage efficiency is imposed by boundary condition assumptions in column-based model structures. Without prognostic spatially resolved models that include subsidence and landscape runoff, the risk for landscape-scale thermokarst-induced abrupt thaw remains highly uncertain.

We used the spatially resolved cryohydrology model Advanced Terrestrial Simulator (formerly Arctic Terrestrial Simulator) (ATS) (16) in a multiscale configuration specific to polygonal tundra (17) to address those uncertainties about the effects of thermokarst formation on polygonal tundra evolution. ATS provides prognostic assessments of overland flow, landscape runoff, and permafrost thermal conditions by coupling the state-of-the-art three-dimensional representations of freezing-soil thermal hydrology with spatially resolved representations of surface energy balance, nonisothermal overland flow, snowpack evolution and thermal processes, and snow redistribution. ATS’s multiscale configuration tessellates the polygonal landscape into many one-dimensional column models, where each column represents the surface expression of a single ice-wedge polygon and the ground beneath (*Methods*). The column submodels use ATS’s detailed representation of surface energy balance, subsurface transport of water and energy, and water accumulation on the surface including phase change. The columns are not directly coupled to each other, but all are sequentially coupled to a common two-dimensional surface system representing overland nonisothermal flow. The effects of microtopography on overland flow are represented through a subgrid model that was developed from microtopography-resolving simulations over individual polygons (18). The subgrid model prevents flow from occurring until the ponded water reaches a threshold amount known as depression storage. We extended ATS’s multiscale configuration for this study to include subsidence and evolution of polygonal ground from low-centered polygons to high-centered polygons. At each time step, the subsidence algorithm collapses computational cells that contain newly melted ice, conserving water, energy, and soil solid mass in the process. The subsidence and consolidation cease when a structurally competent porosity is reached. Depression storage in the overland flow model is reduced as subsidence progresses, which represents the transition from poorly drained low-centered polygons to well-drained high-centered polygons.

The ATS model has been evaluated (16) against multiple laboratory experiments on freezing soils. It has also been carefully compared (19) to multiyear observations of snow depth, suprapermafrost water table depth, depth-resolved soil temperature, and landscape-scale evapotranspiration at our study site (Fig. 1). Those comparisons against observations from a well-characterized site provide a rare degree of confidence in the projections. To further support the model projections, we augment the existing data from our study site with new measurements of ground ice content. Using this data-informed model, we reassess the potential for thaw subsidence to accelerate permafrost degradation and show that the effect is unlikely to lead to large-scale abrupt thaw under the Representative Concentration Pathway 8.5 (RCP8.5) (20) scenario. We also make end-of-century projections of landscape runoff and surface inundation and show that subsidence can help maintain landscape runoff and thus river flows but causes significant drying of polygonal tundra landscapes.

## Results and Discussion

**Fig. 2.**
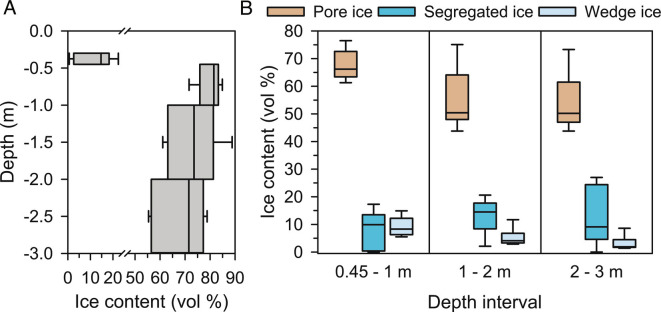
Panel *A* shows the depth distribution of spatially averaged permafrost ground ice content profiles for trough center to trough center cross-sections of six ice-wedge polygons (three low-centered and three flat-centered polygon types) located near Utqiaġvik, AK, which represent key inputs to our modeling. Although the 0.45-m depth boundary corresponds to the average active layer thickness of the sampled polygons, the low ice content above 0.45 m reflects variability in the actual depth of the permafrost table both within and among polygons (*SI Appendix*, Fig. S4). Panel *B* indicates the apportionment of spatially averaged ground ice content into pore ice associated with the soil’s structurally competent porosity, the excess ice fraction associated with segregated cryostructures, and the excess ice fraction contained in ice wedges at each depth interval below 0.45 m. All box plots indicate the median, 20th, and 80th percentiles, with whiskers representing the full range of values (*n *= 6).

New spatially averaged, depth profile estimates of permafrost ice content (volume percent) for transects across entire ice-wedge polygons (from trough center to trough center) were generated by intensive sampling of low- and flat-centered polygons (three of each type) located near the Utqiaġvik study site (Fig. 2). Ice content exceeds the porosity of unfrozen soil (Table 1) throughout the measured depth interval, indicating excess ice that will contribute to soil consolidation and subsidence upon melting. The ice content was highest and least variable just below the active layer and generally declined with depth (Fig. 2*A*), consistent with the observations of ice-rich soil and greater wedge volumes in the upper permafrost throughout much of the Arctic Coastal Plain of Alaska (13, 21). On an average, wedge ice accounted for 11.6% of the total ice content within the 0.45 to 1 m depth interval but only 6.8% and 4.4% at depths of 1 to 2 m and 2 to 3 m, respectively. Hence, the excess ice occurs mostly throughout the soil, as a variety of smaller segregated cryostructures (21), especially below a depth of 1 m (Fig. 2*B*). The volumetric ice contents of individual soil horizons ranged from 55 to 93%, leading to significant variability within and among polygons (*SI Appendix*, Fig. S1). We addressed this uncertainty by conducting simulations that used median, 20th percentile, and 80th percentile ice content profiles.

**Table 1.** Excess ice content (%) versus depth developed from observations from three flat-centered polygons and three low-centered polygons

Depth interval	20th percentile	Median	80th percentile
0.45 m to 1 m	11.4	17.5	19.9
1 m to 2 m	14.0	18.0	22.3
2 m to 3 m	7.1	11.2	26.2

We used the new measurements of ice content and previously published data from our site in the multiscale configuration (17) of ATS to simulate the thermal hydrologic response of a small catchment comprising 468 ice-wedge polygons (Fig. 1*B*) with and without topographic change driven by thaw subsidence. The simulations with topographic change include subsidence at large scales and microtopography changes associated with transition from low- to high-centered polygons (*Methods*). Soil and snow properties are from previous parameter estimation and model evaluation studies (19, 22), which were informed by more than one decade of studies at the site (ngee-arctic.ornl.gov).

After initialization and spinup, the model was forced by meteorological data for 1985 to 2005 from the DayMet data product (23, 24), with bias corrections for relative humidity and snow undercatch (*Methods*). Projections were then run from 2006 through 2098. Meteorological forcing data for that period were projected for the site by adding long-term trends from Community Earth System Model (25) runs from the Coupled Model Intercomparison Project Phase 5 (26) in the RCP8.5 scenario (20) to detrended and looped historical DayMet data for the 31-y period from 1985 to 2015. In this approach, the forcings for early 21st century (2006 to 2036), mid-century (2037 to 2067), and late century (2068 to 2098) are identical except for the long-term secular trend, allowing for direct comparisons for the three periods. Importantly, the forcing data from 2006 to 2015 are very similar to the actual DayMet data, thus allowing for comparisons to site observations.

**Fig. 3.**
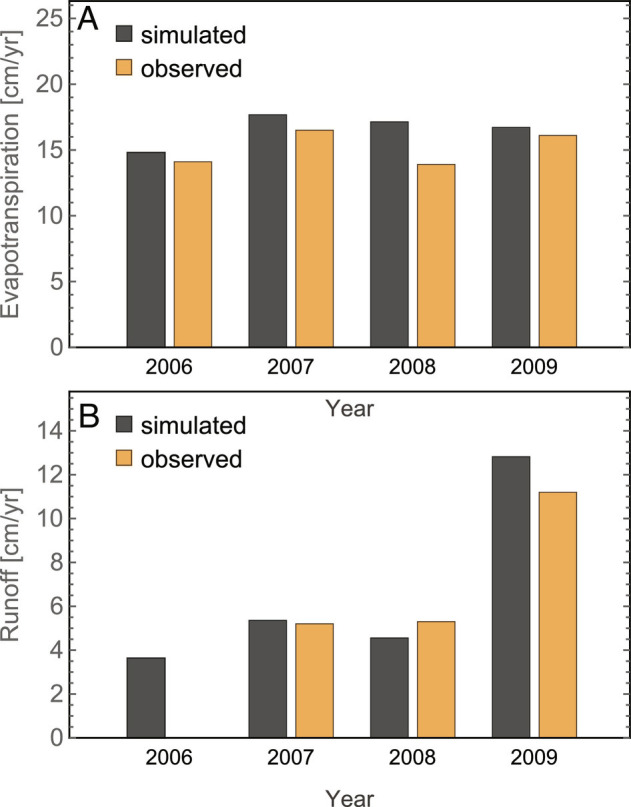
Simulated evapotranspiration (*A*) and runoff (*B*) compared with the observations of Liljedahl et al. (27). Both simulated and observed values are integrated over the summer months, typically June to September. Observed runoff was not available for the year 2006.

Liljedahl et al. (27) analyzed the water budget for the site for the period 2006 to 2009 and provided estimates for runoff and evapotranspiration integrated over the summer periods. Our simulations are in reasonably good agreement with their estimates (Fig. 3) given uncertainties in estimating the components of landscape-scale water budgets, especially evapotranspiration. Summer evapotranspiration is consistently higher in the simulations, with the largest difference occurring in 2008 (about 23%), but integrated over the 4-y period, the difference is less than 10%. For runoff, the agreement is even closer, less than 5% difference integrated over the 3 y when runoff estimates are available. That agreement and earlier successful evaluations of fine-scale ATS simulations (19) against multiple types of observations from this well-characterized site provide a rare degree of confidence in the reliability of the simulations.

**Fig. 4.**
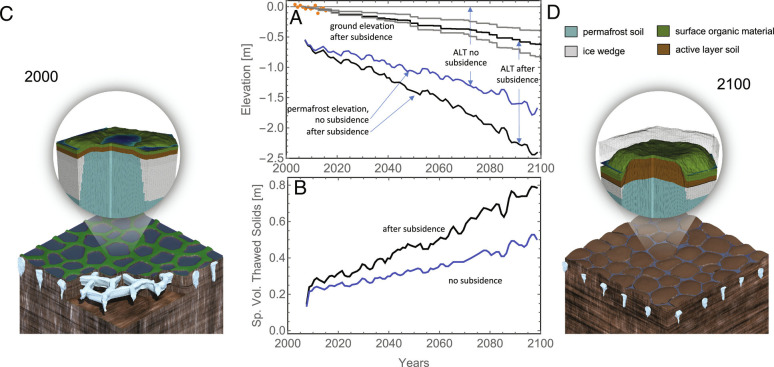
Projections of permafrost thaw and thermokarst development for a polygonal tundra site near Utqiaġvik, AK. Panel *A* shows projected ground elevation and permafrost elevation with (black) and without (blue) subsidence. Three projected ground elevations are shown, corresponding to the median (black) and the 20th and 80th percentiles (gray) of the excess ice distribution. Our reference case using the median ice content is in good agreement with the observed subsidence rates of Streletskiy et al. (28) (orange dots). Shown in Panel *B* is specific volume of thawing soil solids (volume of soil solids experiencing thaw each summer, per unit area) with (black) and without (blue) subsidence. The single-polygon geometric models shown in Fig. 4*C* (early 21st century) and Fig. 4*D* (2100) were constructed by postprocessing our results to illustrate the evolution from poorly drained low-centered polygons to high-centered polygons with well-defined troughs (see text). The larger-scale images are artist renderings of how that microtopographic evolution will affect the landscape.

Results from our century-long projections are shown in Fig. 4. Fig. 4*A* shows projected ground surface elevation after subsidence and permafrost elevation with and without subsidence. Three curves are shown for the ground surface elevation corresponding to the median (black) and the 20th and 80th percentiles (gray) of the ice content profile. Results for the 20th and 80th percentiles are provided to show sensitivity to ice content and should not be interpreted as uncertainty range because the ice content distribution represents spatial variability, rather than parametric uncertainty. Subsidence at year 2100 is 39 cm for the 20th percentile ice content and 87 cm for the 80th percentile, compared to 63 cm for the median depth profile for ice content. The orange dots are ground elevations based on the observed subsidence rates of Streletskiy et al. (28). Simulated subsidence rates for the median ice content profile are in good agreement with their observations. When subsidence is included, the permafrost elevation in 2100 is projected to be approximately 2.4 m below the early-century ground elevation, compared with the initial depth of approximately 0.5 m. Approximately 0.63 m of this elevation change comes from subsidence and the remaining from an increase in the active layer thickness (ALT).

The effect of subsidence on ALT is relatively minor. The ALT for subsiding and nonsubsiding cases nearly overlaps for much of the century and only starts to separate around 2060 (*SI Appendix*). The projected ALT at 2100 is about 1.8 m when subsidence is included and about 1.6 m when subsidence is neglected. That relative insensitivity is the net result of competing effects, as described below. It is important to note, however, that the effect of subsidence on carbon mass thawed each year is larger than the effect on ALT because the thaw front goes through higher-density consolidated soil after the excess ice has melted. In simulations that neglect consolidation and subsidence, the simulated thaw front passes through the same ice-rich soil every year. Because the ice-rich soil has lower carbon density on a bulk volume basis, neglect of consolidation and subsidence underestimates the amount of carbon thawed each summer. The specific volume of thawing soil solids—volume of soil solids experiencing thaw each summer, per unit area—is a more relevant metric than ALT because the amount of soil solids is positively correlated with the quantity of organic carbon that is vulnerable to potential decomposition. The specific volume of thawing soil solids with and without subsidence is shown in Fig. 4*B*. By 2100, the specific volume of soil solids experiencing thaw each summer will be approximately 79 cm when subsidence is included compared to 48 cm when it is neglected.

To better illustrate the effects of the projected subsidence on microtopography, we constructed the geometric models shown in Fig. 4*C* (early 21st century) and Fig. 4*D* (2100) from our results. The early 21st-century geometric model was built from a high-resolution digital elevation map (29) of a single polygon with a representative ice wedge beneath. The geometry of the ice wedge was constrained by the median values for wedge-ice fraction versus depth (Fig. 2*B*). The 2100 geometric model was evolved from that geometry using our subsidence algorithm, the median value of the measured excess ice profile, and a thaw depth of 2.5 m (relative to 2006 ground surface), consistent with our simulations. The geometric model clearly shows the evolution from poorly drained low-centered polygons to high-centered polygons with well-defined troughs that can drain the landscape. The larger-scale images are artist renderings of how that microtopographic evolution will affect the landscape.

The relative insensitivity of ALT to subsidence is the result of multiple competing effects. Subsidence affects the rate of thaw directly through changes in soil conditions and indirectly through changes in microtopography. Subsidence affects soil moisture conditions and amount of surface ponded water directly as soil collapses into water left by melting bulk ice. Thaw rates are dependent on the amount of water present through latent heat effects and through thermal conductivity, which depends on soil water and ice content. The indirect feedbacks caused by changes in microtopography are included in the ATS simulations through dynamic subgrid models that change microtopography and its effect on energy transfer and surface flow (18) as thaw progresses. Differential subsidence creates larger elevation differences between microtopographic lows (e.g., ice-wedge polygon troughs) and highs (e.g., rims and centers of high-centered polygons), leading to thinner snowpack on high points of the microtopography and deeper snowpack in microtopographic low points, the net effect of which is to increase spatially averaged heat transfer with the atmosphere during winter (30). Differential subsidence can also lead to microtopographic high points being exposed above the snowpack for longer periods during spring, which has the effect of decreasing spatially averaged albedo and increasing absorbed shortwave radiation. The net effect of these competing processes is to slightly accelerate thaw as subsidence progresses and alters microtopography.

Our projected subsidence is in line with the results of Nitzbon et al. (10) for Holocene deposits in Northeast Siberia for their well-drained assumption, but our projected ALT is significantly larger (1.8 m versus approximately 1 m) in the warmer climate of the Alaska North Slope. However, our subsidence and ALT are both much smaller than the simulations of Nitzbon et al. that assumed poor landscape drainage (their “waterlogged” case). The two results cannot be directly compared as they are for different sites with different climates. However, differences in surface ponded water are clearly part of the difference, as the waterlogged condition of Nitzbon et al. essentially closes the boundary to lateral flow once subsidence is underway. Since ATS is a spatially resolved model with explicit representation of runoff, surface ponded water is prognostic, spatially variable, and significantly less sensitive to imposed boundary conditions, thus removing a significant uncertainty associated with imposing lateral flow through a boundary condition assumption.

The sensitivity of ALT to surface ponded water has been studied previously (31). For shallow ponded water (<0.30 m), Atchley et al. (31) found thaw depth to be relatively insensitive to the amount of surface water because the competing effects of changes in thermal conductivity and latent heat demands nearly cancel. However, the latent heat demands would clearly dominate once the surface water becomes deep enough. If ponds do not freeze through in the winter, a talik (soil region that remains unfrozen throughout the year) can form and potentially expand. However, abrupt thaw triggered by deep ponded water is unlikely to occur over large areas because of water budget constraints. That is, precipitation in excess of evapotranspiration is small in the study area and in many areas in the Arctic and unlikely to support the formation of spatially extensive deep ponds.

**Fig. 5.**
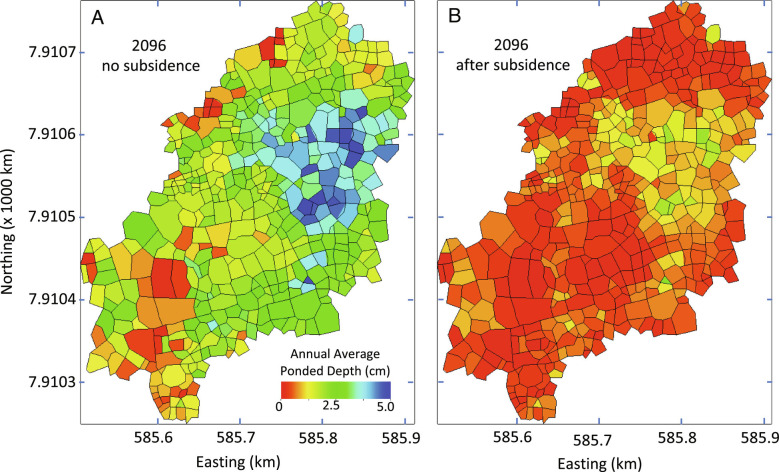
Maps of annual average equivalent ponded depth for the year 2096 without (*A*) and with (*B*) subsidence.

**Fig. 6.**
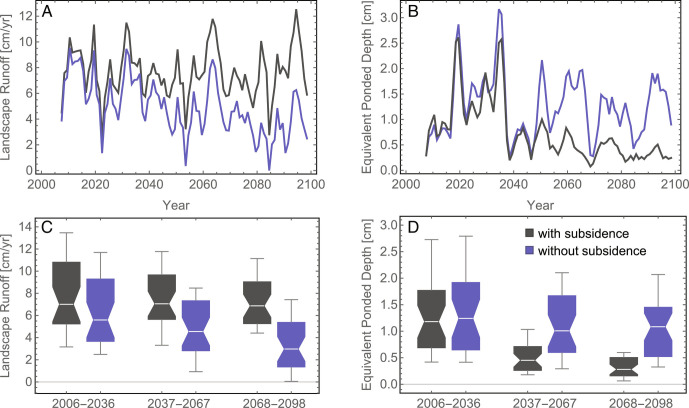
Projections of landscape runoff [cm/yr] and equivalent ponded depth [cm] as time series (*A* and *B*) with (black) and without (blue) subsidence. Results here are spatially and annually averaged values and shown as 3-y moving averages. The box and whisker plots in *C* and *D* show interannual variability in three 31-y time periods representing early, mid, and late 21st century. Each box and whisker plot shows 10th, 25th, 50th, 75th, and 90th percentile values.

A warming Arctic results in increased evapotranspiration, larger thawed soil volumes to store water and buffer precipitation events, and a greater fraction of precipitation falling as rain instead of snow, in addition to the transition from low-centered polygons to high-centered polygons. The net effect of those multiple interacting processes is shown in Figs. 5 and 6. Fig. 5 shows maps of annual average equivalent ponded depth without (A) and with (B) subsidence for the year 2096. When subsidence and the associated change in microtopography are included, the landscape is significantly drier, with most of the domain showing nearly dry conditions. Spatial variability in the equivalent ponded depth is the result of macrotopography and overland flow and illustrates why spatially resolved simulations like those presented here are needed to understand the evolution of permafrost-affected regions.

Fig. 6 shows the evolution of annual runoff (Fig. 6*A*) and spatially averaged equivalent ponded depth (Fig. 6*B*) and quantifies the interannual variability (Fig. 6 *C* and *D*) in those quantities. The year-to-year variability is high, as expected, but long-term trends are clear. The equivalent ponded depth decreases significantly through the 21st century in the subsidence case, but runoff is relatively stable. The opposite trend is evident without subsidence. Evapotranspiration generally increases through the century but at a somewhat reduced rate when subsidence is included (*SI Appendix*). To clearly separate trends from the interannual variability, we divide the simulation period from 2006 to 2098 (inclusive) into early-, mid-, and late-century periods, and show box-whisker plots statistics for each (Fig. 6 *C* and *D*). Because of the way the meteorological forcing for the model was constructed, each of these 31-y periods has the same meteorological forcing except for the long-term secular trend, thus allowing for direct comparison. With subsidence, there is a strong downward trend in surface water ponded depth, but no significant trend in runoff. Landscape drying is strongest in dry years. Late-century surface water in dry years (25th percentile years) is approximately 23% of the early-century value, suggesting significant loss of wetland breeding sites for migratory bird species. Importantly, drying and runoff trends are reversed in the absence of subsidence, underscoring the need to include that process in site-, regional-, and panArctic-scale models.

## Conclusions

Our spatially resolved, physics-based simulations of a well-characterized site on the Alaska North Slope suggest that thermokarst formation will have relatively modest effects on ALT. The ALT projected for 2100 in the strong-warming RCP8.5 scenario is 1.8 m when subsidence is included compared to 1.6 m when it is neglected. The projected subsidence is about 63 cm.

Previous modeling efforts by Nitzbon et al. (10) addressed potential thaw acceleration from thermokarst formation and showed high sensitivity to landscape drainage efficiency, which was imposed through prescribed boundary conditions in their study. Our projections fall between their well-drained and waterlogged cases but are much closer to their well-drained case. In the spatially distributed models used here, water flows across the landscape are simulated explicitly. Runoff from the modeled catchment is thus prognostic and relatively insensitive to assumed boundary conditions, which removes a key uncertainty. Our results suggest that the most dramatic of the scenarios in the Nitzbon et al. simulations—their poorly drained (waterlogged) scenario wherein thermokarst formation triggers rapid permafrost thaw and large increases in thaw-exposed soil organic carbon—is unlikely to occur over large areas.

Although our simulations suggest that subsidence will only have minor effects on the projected ALT, they show significantly greater impact on thaw-exposed soil solids. Specifically, the amount of soil solids thawing each summer is roughly 65% higher in the subsiding case compared to the nonsubsiding case because the thaw front passes through denser consolidated soils after subsidence. Increases in thaw-affected soil solids imply increases in thaw-affected soil organic carbon, but quantifying the impact on soil organic carbon stocks in permafrost regions requires additional assumptions and models, which is left for future studies. Nevertheless, these results do emphasize the need to include subsidence and soil consolidation in Earth system models that address carbon cycle dynamics in permafrost regions.

Our results show significant effects of thermokarst formation on landscape runoff and ponded surface water. Simulations with thaw subsidence show strong trends to drier conditions throughout the century as landscape drainage is improved by the transition from low-centered polygons to high-centered polygons, leaving less water stored on the landscape. Drier tundra conditions suggest a loss of wetland breeding sites for migratory bird species. In contrast, landscape runoff is maintained in the face of increased evapotranspiration but only when subsidence is included, suggesting that microtopography changes caused by thaw subsidence will help support Arctic streamflow, thereby providing aquatic ecosystems a degree of resiliency in a warming climate.

We chose site-specific simulations of a well-characterized site to evaluate the effect of thermokarst formation because of existing data and previous model calibration and model evaluation work. Our multiscale simulations are in good agreement with observed evapotranspiration and landscape runoff for the period 2006 to 2009. They also agree with the estimated subsidence rates in the current climate. Those model evaluations in combination with our previous model evaluation work provide a unique degree of confidence in the model projections for a given warming scenario. The site modeled is typical of low-relief polygonal Arctic tundra, and the qualitative conclusions about the consequences of thermokarst formations are expected to apply broadly to that type of landscape, which represents large swaths of the Arctic. However, the site specificity also means that quantitatively scaling to large regions to evaluate large-scale impacts will require additional model development, for example by using similar modeling studies to improve parameterizations in Earth system models.

## Materials and Methods

The simulations used the ATS (32), in a multiscale configuration (17) specific to polygonal tundra. That multiscale model was updated for this work to include the effects of bulk subsidence and local microtopography changes induced by thawing ground ice (33). Ground ice content, a key input to the subsidence model, was synthesized from spatially and vertically resolved profile estimates across six ice-wedge polygons (four in the immediate vicinity of the study area and two located <2.5 km to the south).

### Ground Ice Measurements.

Two-dimensional profile estimates of ice content to 3-m depth (Dataset S1) (34) were derived from a total of over 350 samples collected from transects across each ice-wedge polygon using methods described by Ping et al. (35). Soil pits, trenches, and cores along the transects were used to describe, sample, and map the cross-sectional stratigraphy of soil horizons and ice wedges, from trough center to trough center (*SI Appendix*, Fig. S3). The upper permafrost boundary was identified by the examination of cryostructures and their positioning (36, 37). The average water content (volume percent) of each soil horizon was estimated from the measurements of gravimetric water content and dry bulk density for multiple samples per horizon and, below the permafrost table, converted to volumetric ice content using a density of 0.917 g cm^−3^. Areas occupied by ice wedges were assigned volumetric ice contents of 100%. The ice contents of soil horizons and wedge ice were weighed by their cross-sectional areas to determine spatially averaged profile estimates of ice content across the entire polygons at four depth intervals (0.3 to 0.45 m, 0.45 to 1 m, 1 to 2 m, and 2 to 3 m), where the boundary at 0.45 m corresponds to the average ALT of the sampled polygons (*SI Appendix*, Fig. S4).

### Estimation of Excess Ice Content.

The excess ice content versus depth is a key input to the subsidence algorithm, as it directly controls the rate of subsidence. The excess ice fraction was estimated from the ice-content measurements as the difference between the measured ice content and the structurally competent porosity. The structurally competent porosity was estimated by first classifying observed soil horizons according to the four types with increasing amounts of visibly organic components (mineral, mineral/organic, organic/mineral, and organic). Porosity determined from the water content (volume percent) of the observed horizons located above the permafrost table (generally from the intermediate 0.15 to 0.3 m depth interval within the active layer) was then assumed (after conversion to volumetric “ice” content and spatial averaging) to represent the structurally competent porosity for each soil type. Structurally competent porosity for each depth interval below the permafrost table was then determined as an average of the porosities of each soil type weighed by the relative cross-sectional area of each type within the depth in question. We calculated excess ice content this way for each of the flat- and low-centered polygons (Dataset S1, ref. (34)) and used the median as our reference-case initial condition. The median, 20th percentile, and 80th percentiles are shown for 3 depth intervals in Table 1. We further used the estimates of structurally competent porosity, excess ice content, and the area occupied by ice wedges within polygon cross-sections to partition the total spatially averaged ice content of each polygon into three components for each depth interval below 0.45 m. First, the amount of ice held within the soil’s structurally competent porosity was assumed to represent the pore ice fraction. The wedge-ice fraction was calculated by multiplying the area occupied by ice wedges within each polygon cross-section times an assumed volumetric ice content of 100%. The fraction of excess ice associated with segregated cryostructures was calculated as the difference between excess ice and the wedge-ice fraction.

### Processes Represented in ATS.

The cold-region hydrology processes represented in ATS are summarized here and described in detail elsewhere (14, 16). They include surface energy and water balances, snow thermal processes, snow compaction and redistribution, two-phase (ice/liquid) overland flow, two-phase variably saturated subsurface flow, heat advection in the surface and subsurface, and subsurface heat conduction. Constitutive relationships are highly nonlinear and strongly couple the mass and energy conservation equations.

Subsurface water movement in variably saturated conditions is represented by a two-phase (ice, liquid) extension of Richards equation (i.e., with a passive gas phase). Constitutive relationships among temperature, liquid pressure, unfrozen water content, and total water content are based on thermodynamic constraints (generalized Clapeyron equation) and standard soil moisture/pressure relationships measured in unfrozen conditions (38). In this physics-based approach, empirical “freezing curves” that relate unfrozen water content to temperature are not required. Shallow surface flow is represented by the diffusion wave equation coupled to heat transport equations and accounting for water freezing and thawing. Previous sensitivity analyses (17, 19) show that strong coupling between surface and subsurface flow systems in integrated models makes the surface flow solution relatively insensitive to the choice of Manning’s coefficient *n* in the diffusion wave equation provided *n* is sufficiently small; we use *n* = 0.1 *s*·*m*^−1/3^, which is in the insensitive range identified by those studies.

Total evapotranspiration (ET) in the cold-region configuration of ATS results from the surface energy balance, which includes latent and sensible heat transfers and surface radiative balance. ET is not partitioned between evaporation and transpiration, effectively relocating transpiration losses from the root zone to the near-surface soil region. This is an appropriate approximation for our study site which is dominated by evaporation losses (39) and for tundra landscapes in general that are dominated by nonvascular mosses and shallow-rooted grasses and sedges.

The constitutive relationships and the soil thermal hydrology models in ATS have been evaluated against multiple laboratory experiments on freezing soils (16, 38, 40). The permafrost configuration of ATS has also been successfully evaluated against snow depth, vertically resolved soil temperatures at multiple locations, water table elevation, and evapotranspiration measurements over a 3-y period (19).

### Multiscale Configuration of ATS.

The multiscale configuration of ATS tessellates the landscape into many one-dimensional permafrost column models, each associated with an ice-wedge polygon (*SI Appendix*, Fig. S5). Each column submodel includes a representation of two-phase soil thermal hydrology, snow thermal processes including compaction, surface water ponding and evaporation, and surface radiative energy balance. The columns are not directly coupled to each other, but all are sequentially coupled to a common two-dimensional surface system representing overland nonisothermal flow. This spatial configuration was motivated by the results of fine-scale simulations. Those simulations showed that significant variations in temperature and moisture content among center, rim, and trough positions in ice-wedge polygons are relatively short lived. On seasonal time scales, lateral subsurface flow and heat transport are sufficient to equilibrate temperature and water table across each polygon (17), which makes it reasonable to use ice-wedge polygons as the basic mesh unit in decadal simulations. A second assumption—that overland flow between polygons dominates over subsurface lateral flow—has been checked by fully three-dimensional simulations and found to be a good approximation for catchment-scale simulations (17).

Although the effects of microtopography below the scale of the ice-wedge polygon are neglected in the subsurface, they are important for overland flow and are included through subgrid models developed from fine-scale simulations (18). As described previously, the subgrid model modifies the accumulation term in the conservation equation for ponded water and the overland flow law to account for important effects of subgrid microtopography. The modified flow law includes depression storage and obstruction drag of the rough land surface. Depression storage is implemented as a threshold value for water depth below which overland flow does not occur. It captures the effect of stagnant water ponding in polygon centers. Importantly, depression storage and other elements of the subgrid model are strongly dependent on polygon morphology. In general, high-centered polygons have less depression storage than that of low-centered polygons.

### Representation of Thaw Subsidence and Microtopographic Change.

We extended our multiscale model for this study to account for thaw subsidence and its effect on microtopography. Bulk subsidence—subsidence averaged over each ice-wedge polygon—is represented using an algorithm similar to that introduced by Painter et al. (41). In this approach, we define a *structurally competent porosity*, which is the porosity that the initially frozen soils will consolidate to after thawing. Ice-filled porosity above the structurally competent porosity is excess ice. When a grid cell in the ice-rich zone begins to thaw, the cell volume is decreased by the volume of the melted excess ice to account for the soil consolidation, and the porosity is updated to conserve soil mass. This consolidation is evolved in time fully coupled with the mass and energy conservation equations. As a result, when a cell collapses, there is a compensatory flux out of the collapsing cell, typically to the cell immediately above. The consolidation process is halted once the porosity reaches the structurally competent porosity. Elevation of each cell in the surface mesh where the surface flow is represented is then updated accounting for the subsidence over all cells in the associated soil column. The formation of new ice lenses as the soil refreezes each winter and the resulting frost heave are neglected. The algorithm is likely an upper bound on the rate of subsidence. However, the increase in excess ice associated with the newly formed ice lenses will contribute to additional subsidence in subsequent summers, partially compensating for the frost heave. The redistribution of water due to ice lens formation will also have minor effects on the spatial profile of thermal conductivity, but that is a second-order effect.

The effect of subsidence on subgrid microtopography is accounted for by making the subgrid model dynamic. The subgrid model accounts for the effects of microtopography on overland flow and includes four parameters: microtopographic relief, excluded soil volume, depression depth, and flow drag exponent. Details of this model can be found in the study by Jan et al. (18). The microtopographic relief and excluded soil volume are purely geometric quantities and were calculated directly from high-resolution digital elevation models (DEMs). The depression depth and flow drag exponents cannot be directly estimated from DEMs but have been inferred by calibration to microtopography-resolving overland flow simulations. Jan et al. (18) analyzed seven polygons this way. Based on those results, we identify typical values for low- and high-centered polygons (*SI Appendix*, Table S1). Low-centered values are assigned to polygons that have not experienced subsidence, high-centered values are assigned to polygons that have experienced subsidence of 40 cm or more, and linear interpolation is used between those end member cases. All the four subgrid parameters are interpolated this way. However, Jan et al. (17) identified greater sensitivity of hydrograph to depression depth and drag exponent parameters than the microtopographic relief and soil excluded volume.

### Model Setup and Spinup.

The model domain shown in Fig. 1 was developed from a high-resolution lidar-derived DEM (29). We first selected the catchment outlet to coincide with the location of observed flowing water during snowmelt. The contributing area to that location was delineated from the DEM. The 468 polygons in the catchment were then delineated by hand and classified as high centered or low centered from visual inspection. The resulting surface mesh was then extruded into the subsurface to form the three-dimensional primal mesh. The extruded mesh has 80 cells in the vertical direction with variable vertical spacing starting at 2 cm for the topmost cell and gradually increasing to ~13 m at the domain bottom 40 m below the surface. Five soil layers were used in the subsurface: a 2-cm moss layer; a peat layer with thickness 18 cm for low-centered polygons and 8 cm for high-centered polygons; a mineral soil layer between the peat and 0.5 m depth, the approximate depth of the current-day active layer; a 2.5-m ice-rich layer below that; and finally mineral soil at depth. The ice-rich layer was further divided into three-depth intervals as discussed below. The individual column meshes and the surface mesh used in the multiscale simulation were extracted from the primal mesh at runtime.

Bottom boundary conditions for the simulation are no water flow and specified temperature (−6 °C) based on borehole measurements in the area (42). Boundary conditions for the overland flow domain are zero head at the defined outlet and no-flow elsewhere. The meteorological forcings including air temperature, rain precipitation, snow precipitation, humidity, and incoming short-wave radiation were taken from DayMet (23, 24). Snow precipitation was enhanced by a factor of 30% to account for snow undercatch (14). Humidity from DayMet is unrealistically low during dry periods at this site and was truncated at 70% to avoid those unrealistic values. Incoming long-wave radiation is not available from DayMet and was developed from well-established correlations with air temperature and humidity (43). Model spinup used highly smoothed version of these meteorological forcings calculated by average values for each day of the year to obtain a typical year. Projections starting in the year 2006 used looped historical DayMet data for the 31-y period from 1985 to 2015 with an added trend. The DayMet temperature was first detrended and then combined with a long-term temperature trend from Community Earth System Model (25) runs from the Coupled Model Intercomparison Project Phase 5 (26) in the RCP8.5 scenario (20); see *SI Appendix*, Fig. S6. In this approach, the forcings for early 21^st^ century (2006 to 2036), mid-century (2037 to 2067), and late century (2068 to 2098) are identical except for the long-term secular trend in air temperature (and incoming long-wave radiation), allowing for direct comparisons for the three periods.

A two-step spinup process was used to initialize the simulation. The first step was to simulate one high-centered polygon and one low-centered polygon using the looped “typical year” DayMet data as forcing. This step placed each column in a cyclic steady state. The results of those simulations were then mapped into the 3D primal mesh to provide an initial condition for the second spinup step. The second spinup step used that 3D state as initial condition and was forced by historical DayMet data for the period 1985 to 2005. Results from that spinup step were then used as initial conditions for the projections.

Soil and surface parameters needed in the simulation were taken from previous modeling studies of the site (14, 19, 22) that focused on parameter estimation and model evaluation against multiple types of observations.

## Supplementary Material

Appendix 01 (PDF)

## Data Availability

1. Model Archive (https://ngee-arctic.ornl.gov/data/pages/NGA288.html) (44); 2. Field Data; and 3. ATS Source Code data have been deposited in: 1. Next Generation Ecosystem Experiments Arctic Data Collection (https://ngee-arctic.ornl.gov/data/pages/NGA178.html) (32); 2. ESS-DIVE (https://data.ess-dive.lbl.gov/view/doi:10.15485/1876898) (34); and 3. Github (https://github.com/amanzi/amanzi/releases/tag/ngee_ism_v2) (33).
