# Supplementary material for: Drying of tundra landscapes will limit subsidence-induced acceleration of permafrost thaw
Source: Proc Natl Acad Sci U S A. 2023 Feb 13;120(8):e2212171120. doi: 10.1073/pnas.2212171120 (PMC9974406; doi:10.1073/pnas.2212171120)
Supplement: Supplementary file 1 — Appendix 01 (PDF) [file pnas.2212171120.sapp.pdf]

Supplementary Information for

Drying of tundra landscapes will limit subsidence-induced acceleration of permafrost thaw

Scott L. Painter, Ethan T. Coon, Ahmad Jan Khattak, and Julie D. Jastrow

To whom correspondence should be addressed:

Scott L. Painter, Oak Ridge National Laboratory

Email: paintersl@ornl.gov

This PDF file includes:

Supplementary text

Figures S1 to S8

Table S1

Legends for Datasets S1 to S3

SI References

Other supplementary materials for this manuscript include the following:

Datasets S1 to S3

Supplementary Information Text

S.1 Estimation of excess ice content for each ice-wedge polygon

In our forward simulations, we used a stylized depth profile of soil properties that is consistent with our previous work on parameter estimation and model evaluation and broadly consistent with soil measurements described above. The structurally competent porosity was assumed to be 56% in the forward simulations. Independent of that model input, we need to estimate the structurally competent porosity for each polygon for the purposes of turning ice content (a field observation) into excess ice content (a model input). To that end, soil samples from the active layer and from the permafrost zone were classified into 4 soil types: mineral, mineral/organic, organic/mineral, and organic. Porosity associated with each type from the active layer and relative spatial abundance of those soil types for several depth intervals in the permafrost zone were used to estimate the structurally competent porosity. Specifically, we estimated $\phi_{sc,i}$, the structurally competent porosity in each depth interval i, as

$$\phi_{sc,i} = \left(f_{min,i}\phi_{min} + f_{mo,i}\phi_{mo} + f_{om,i}\phi_{om} + f_{org,i}\phi_{org}\right)/\left(f_{min,i} + f_{mo,i} + f_{om,i} + f_{org,i}\right)$$

Here $\phi_{min}, \phi_{mo}, \phi_{om}$ and ϕ_{org} are the structurally competent porosities for mineral, mineral/organic, organic/mineral, and organic soils, which were approximated by measurements from the current-day active layer. Note the relative abundances in each soil horizon $f_{min,i}, f_{mo,i}, f_{om,i}$ and $f_{org,i}$ in general do not sum to 1 because of the presence of wedge ice. Excess ice content for each depth interval of each polygon was then calculated by subtracting the structurally competent porosity from the measured volumetric ice content. Calculations and results can be found in supplemental dataset S01.

S.2 Calculation of specific volume of soil solids thawed

The change in active layer thickness in the future climate does not directly quantify the additional amount of soil material that will thaw each summer because consolidation of ice-rich soil after thawing significantly increases the volume fraction of soil solids relative to the present-day unthawed value. The specific volume of soil solids thawed $1 - \phi$, where ϕ is porosity, can be estimated from the active layer thickness, excess ice content, and structural porosity. With subsidence, the late-century active layer thickness is projected to be about 1.8 m. With our assumption of a structurally competent porosity of 56%, the specific volume of soil solids thawed in the late-century summers relative to that thawed in the summers of the current climate is estimated as (1.8 m – 0.5 m)*(1 – 0.56) = 0.57 m. Here 0.5 m is the approximate active layer thickness in the current climate.

If subsidence is neglected, the active layer thickness is projected to be about 1.6 m at the end of the century and we need to use the total ice content instead of the structural porosity. Without subsidence, we estimate the specific volume of soil solids thawed in the late-century summers relative to present day to be (1.6 m – 1.0 m)*(1-0.74) + (1.0 m – 0.5 m)*(1-0.735) = 0.29 m. Here 0.735 and 0.74 are the total ice content used in the simulation for the depth intervals 0.5 m to 1.0 m and 1.0 m to 1.6 m.

Time-dependent calculations of the specific volume of soil solids in absolute terms (i.e. not relative to the early century climate) can be found in the mathematica notebook Fig4B.nb

Supplemental Figures and Tables

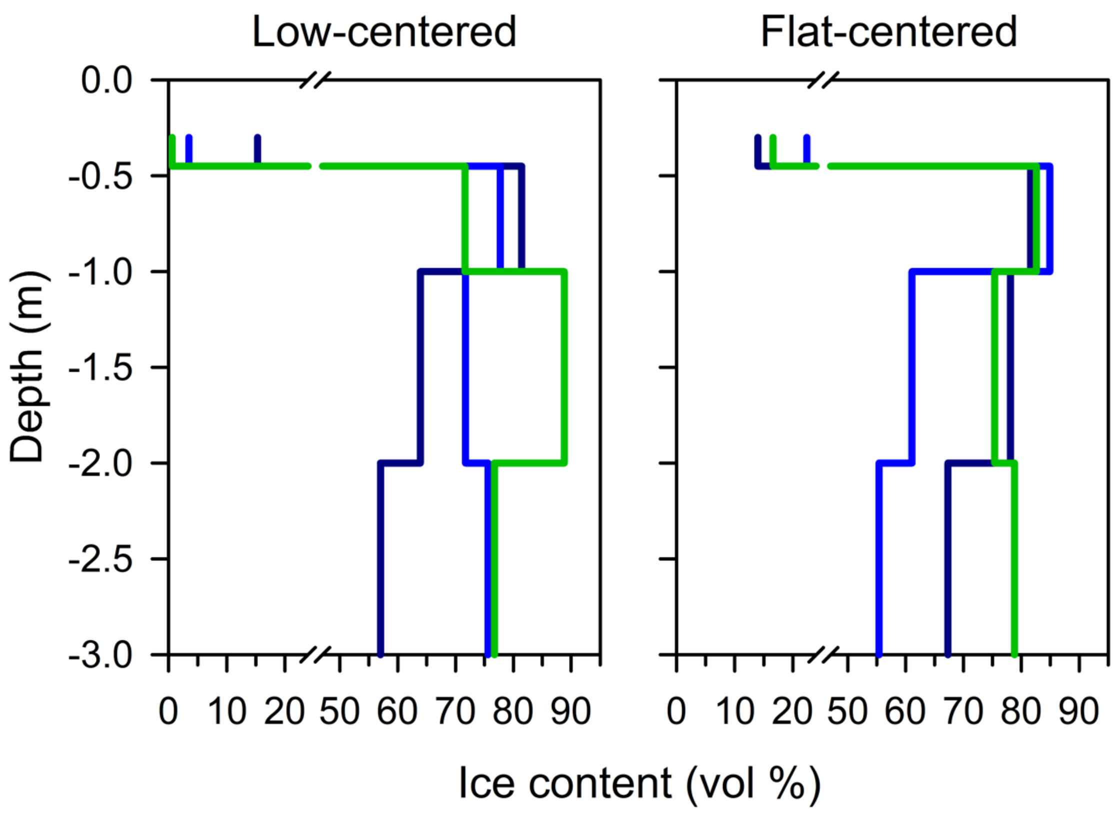

Figure S1. Depth profiles of spatially averaged permafrost ground ice content determined for trough center to trough center cross sections of three low-centered and three flat-centered ice-wedge polygons located near Utqiaġvik, AK. Although the 0.45-m depth boundary corresponds to the average active layer thickness of the sampled polygons, the low ice content above 0.45 m reflects variability in the actual depth of the permafrost table both within and among polygons (Fig. S3).

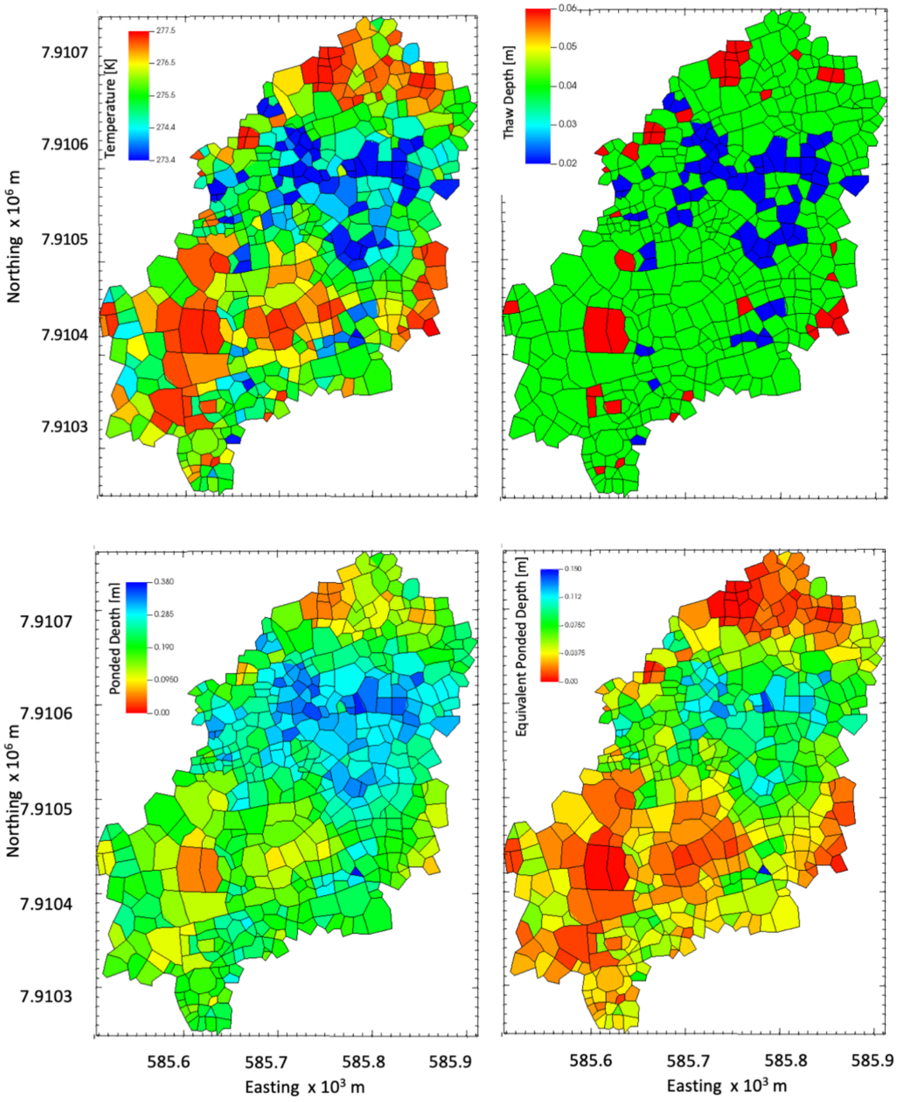

Figure S2. Snapshot of spatially resolved ATS simulations at day 129 of year 2098 showing spatial heterogeneity in surface temperature, snow depth, ponded depth, and equivalent ponded depth during the snowmelt period. The two ponded depth variables are different because of subgrid variability in topography. The equivalent ponded depth is a volumetric quantity (volume of water per unit surface area) while the ponded depth is the simulated water level in water filled troughs. These first-of-a-kind spatially explicit simulations are prognostic for overland flow and landscape runoff, thus reducing a major uncertainty in projections of permafrost thermal hydrology and subsidence.

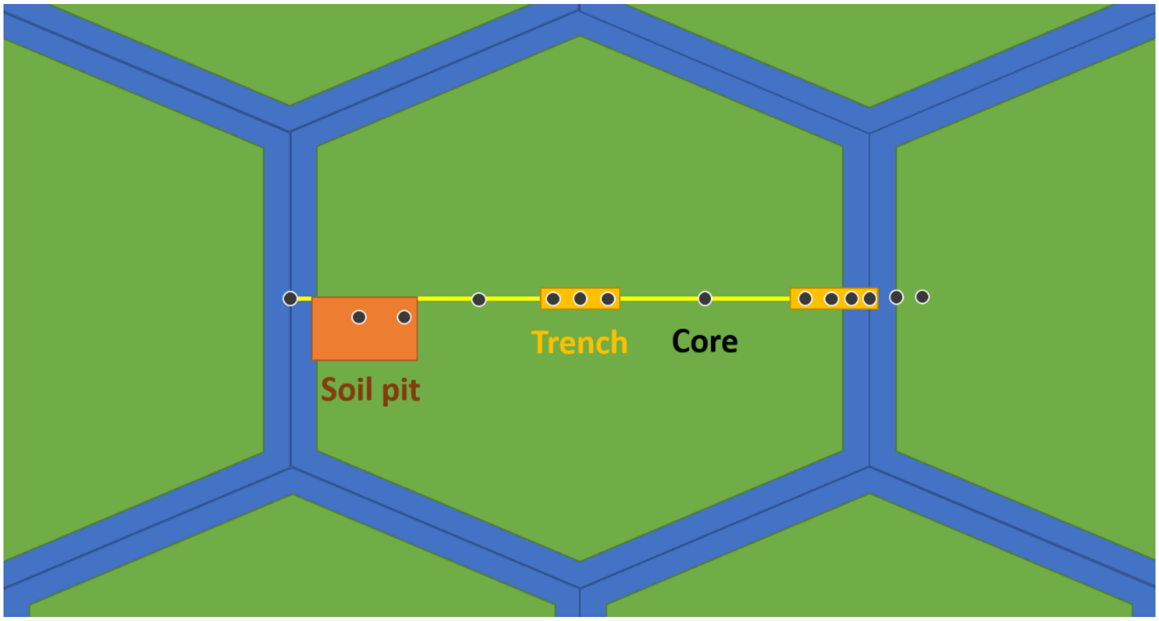

Figure S3. Generalized illustration of the polygon sampling approach used to generate and map two-dimensional cross sections of soil horizon stratigraphy and ice wedge dimensions. The yellow line indicates the sampling transect laid out from trough center to trough center. Soil pits (usually 2-m long by 1-1.5 m deep) were excavated with shovels and jackhammers and were typically placed from the edge of the trough over the rim towards the center of the polygon to capture soil cryoturbation associated with ice wedge growth over time. Frozen soil cores were taken with a SIPRE corer from the bottom of excavated pits to depths of up to 3 m from the surface. Trenches were cut with spades to examine and sample the stratigraphy of thawed surface soils, and SIPRE cores were taken from the bottom of the trench to sample frozen soil at depths of up to 2.5-m. For individual cores, a small block of thawed surface soil was first cut and removed with a spade for sampling, followed by coring of the frozen soil beneath. A number of closely spaced individual cores were taken to determine the size and shape of ice wedges. The specific sampling design applied to each polygon varied depending on its diameter, hydrologic conditions, and the amount of spatial variation in stratigraphy observed during sampling.

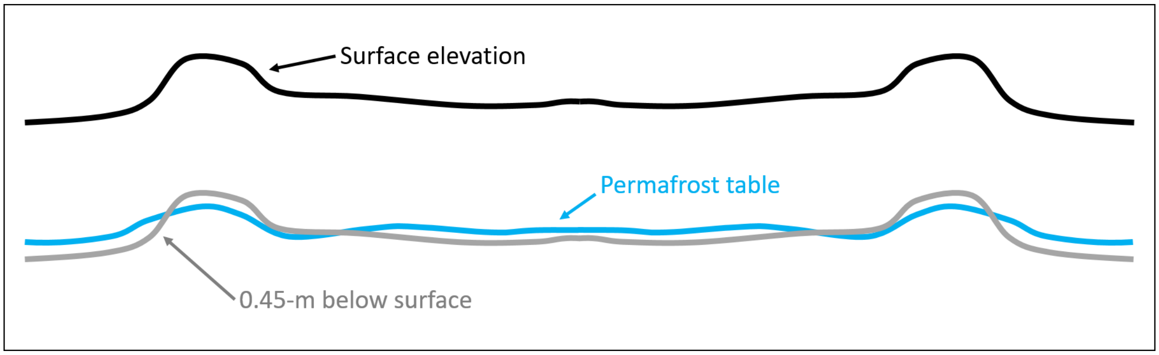

Figure S4. Conceptual diagram illustrating how the actual position of the permafrost table located at a spatially averaged depth of 0.45 m does not correspond directly with a measured depth of 0.45 m below the surface elevation of an ice-wedge polygon. This lack of direct correspondence within a given polygon, coupled with variation among polygons, explains the existence of a small amount of ground ice above the average thickness of the active layer as shown in Figs. 2 and S1. Similarly, a small proportion of active layer horizons occurring below 0.45 m, slightly lowers the spatially averaged permafrost ground ice content of entire polygon cross sections at the 0.45-1 m depth interval compared to the ice content calculated on the basis of permafrost area within this depth interval (see supplemental Dataset S01).

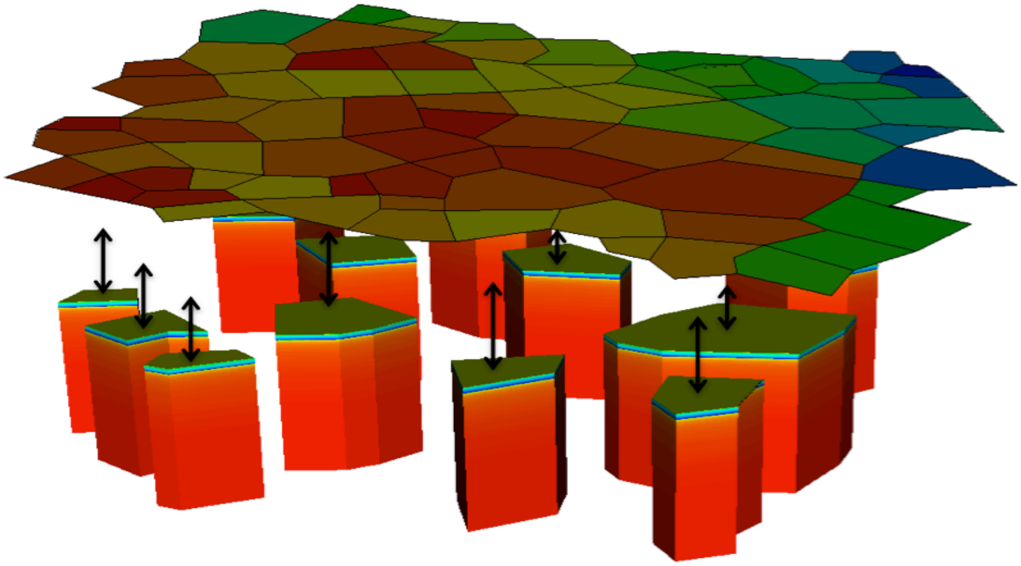

Figure S5. Model structure for ATS configured as an intermediate-scale model for polygonal tundra hydrology. Each vertical 1-D column represents tundra associated with a single ice-wedge polygon and includes a column of soil, a single surface grid cell for the surface water system, and a surface energy balance. The two-dimensional surface mesh is used for the solution of overland flow. The columns are advanced independently of each other, subcycling as needed. At predefined synchronization times (every 6 hours in this case), water temperature and ponded depth on the column surface cells update the surface system. Water and energy are then redistributed on the surface system by advancing that system. The surface cells of the columns are then updated, and the process repeated.

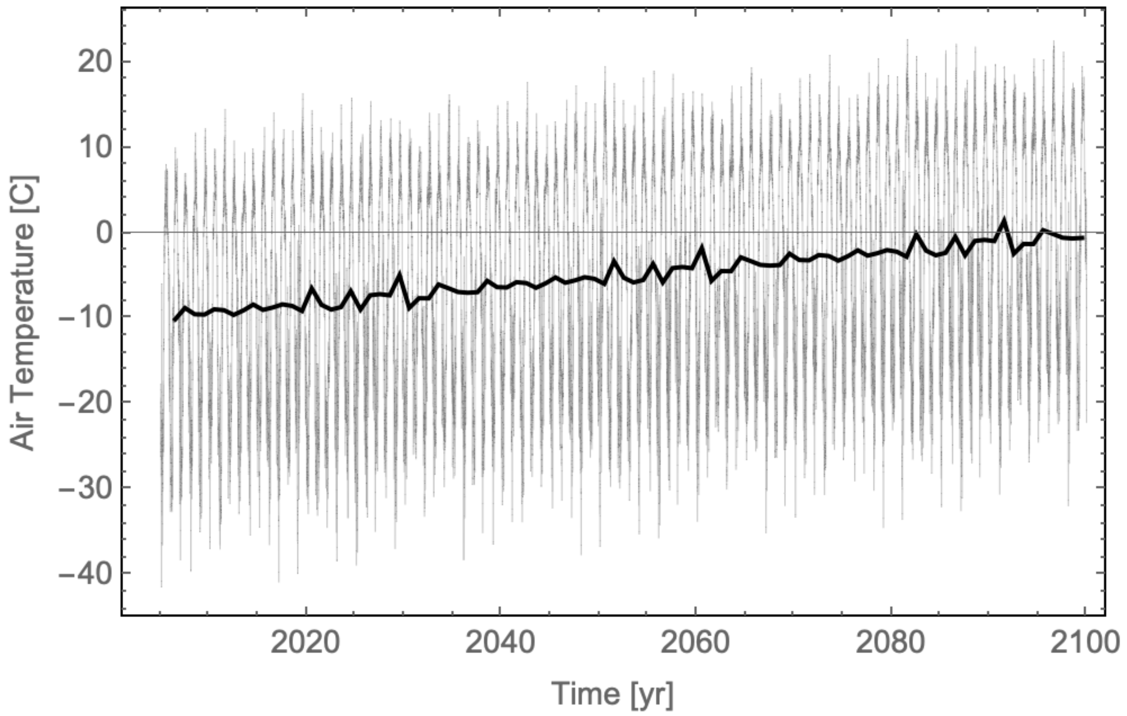

Figure S6. Air temperature projections developed by adding looped and detrended DayMet (1,2) data (31-year record) to long-term trend extracted from climate model projections (see text for details). The heavy curve is annual average. The light line is the daily value used as forcing to ATS.

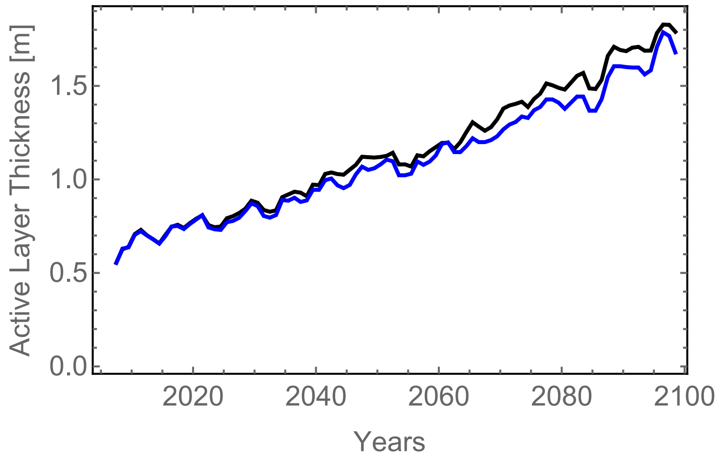

Figure S7. Active layer thickness versus time with and without subsidence. Blue and black curves are without and with subsidence, respectively.

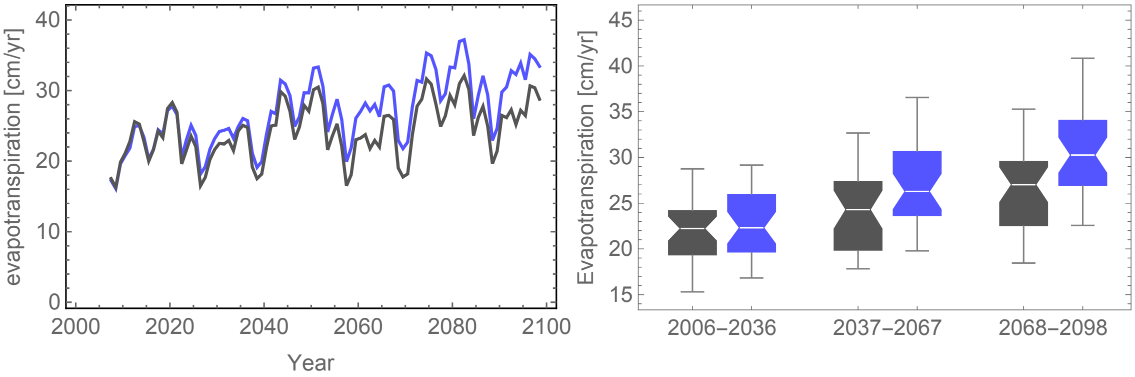

Figure S8. Left panel is evapotranspiration versus time. Right panel shows interannual variability in evapotranspiration for three time periods. Blue is without subsidence, black is with subsidence.

Table S1. Parameters in our subgrid model for the effects of microtopography on overland flow in polygonal tundra (3) for low-centered and high-centered polygon. For this work we made the subgrid flow model dynamic by transitioning between low-centered and high-centered parameters as the thaw front progresses into previously frozen soil. The depression storage parameter represents the amount of water that can pond on the surface before flow initiates and is the most important subgrid parameter. See Jan et al. (3) or parameter definitions.

	Depression depth [m]	Microtopographic relief [m]	Specific excluded volume [m]	Obstruction drag exponent [-]
Low-centered	0.15	0.4	0.2	7
High-centered	0.05	0.4	0.2	1

Supplemental Datasets

S01 Spatially Averaged Ice Contents of Ice-Wedge Polygon Cross-Sections to 3-m Depth, July 2013, Utqiagvik, Alaska. doi:10.15485/1876898 accessed via https://data.ess-dive.lbl.gov/datasets/doi:10.15485/1876898

S02 Model Archive

https://doi.org/10.5440/1876200 accessed via ngee-arctic.ornl.gov/data

Includes

Simulation results for ATS projections.xlxs Excel workbook with daily simulation results for projections compiled on the surface mesh. Time is in days measured from beginning of 1985.

BarrowProjections.tar.gz Model archive for “Drying of tundra landscapes will limit subsidence-induced acceleration of permafrost thaw”. Input files for spinup and projections and selected checkpoint files for model restarts.

Figs3and6AB.nb Fig4A.nb Fig4B.nb Figs6CD.nb Mathematica Notebooks for processing spatially averaged simulation results into figures.

Utqiagvik AK IWP Ice Contents - Calculation of Excess Ice_11-27-2022.xlsx Excel workbook calculating the model input excess ice content from spatially averaged ice contents

S03 ATS Source Code

The version of ATS used for this work can be found at https://github.com/amanzi/amanzi/releases/tag/ngee_ism_v2

SI References

1. M. M. Thornton *et al.* (2020) Daymet: Daily Surface Weather Data on a 1-km Grid for North America, Version 4. (ORNL Distributed Active Archive Center).
2. P. E. Thornton *et al.*, Gridded daily weather data for North America with comprehensive uncertainty quantification. *Scientific Data* **8**, 190 (2021).
3. A. Jan, E. T. Coon, J. D. Graham, S. L. Painter, A Subgrid Approach for Modeling Microtopography Effects on Overland Flow. *Water Resources Research* **54**, 6153-6167 (2018).
